# Sarcoidosis in the Genomic Era: From Genetic Drivers to Tailored Therapies

**DOI:** 10.1007/s11882-025-01222-9

**Published:** 2025-09-30

**Authors:** Natalia V. Rivera, Dominique Israël-Biet

**Affiliations:** 1 Division of Immunology and Respiratory Medicine, Department of Medicine Solna, Karolinska Institutet, Karolinska University Hospital, Solna, and Center for Molecular Medicine, Karolinska University Hospital, Stockholm, Sweden; 2 Université de Paris, APHP, Service de Pneumologie, Centre de Compétences Maladies Pulmonaires Rares, Hôpital Européen Georges Pompidou, Paris, France

**Keywords:** Sarcoidosis, Human leukocyte antigen, Pharmacogenetics, Biomarkers, Endophenotypes, ILD, Pharmacogenetics, Pharmacogenomics

## Abstract

**Purpose of Review:**

This review aims to synthesize existing literature on the genetics of sarcoidosis, including the genetic architectures associated with various clinical phenotypes, as well as current treatment options. It will also examine studies on phenotyping and endophenotyping sarcoidosis, along with offering new perspectives into pharmacogenetics and pharmacogenomics. The latter remains largely unexplored, which could potentially lead to new opportunities and further the goals of precision medicine.

**Recent Findings:**

Genetics and genomics have provided new insights into the study of sarcoidosis. According to current literature, there are variations in the genetic structure of sarcoidosis when categorized by phenotypic definitions. A common element among these findings is the *HLA-DRB1* gene, which is associated with many autoimmune diseases. Genetic analysis is a valuable tool for identifying patient groups based on their genetic profiles, offering an opportunity to classify patients for targeted treatment approaches.

**Summary:**

Genetics can provide valuable insights that, when combined with other omics disciplines, can aid in diagnosing and managing sarcoidosis and help discover new disease biomarkers. Genetics improve the detection of sarcoidosis endophenotypes, and the combination of pharmacogenetics and pharmacogenomics will support the use of appropriate treatments and help eliminate unnecessary therapies in patients with specific genetic susceptibility.

## Introduction

Sarcoidosis is a complex, multisystem granulomatous disease that is immunologically driven, predominantly affecting the lungs but capable of involving nearly any organ. Despite being first described over a century ago, the etiology, pathogenesis, and optimal management strategies for this condition remain poorly understood. Moreover, sarcoidosis is a heterogeneous disease characterized by the presence of non-caseating granulomas within the affected organs. Its variability stems from an insidious onset, likely precipitated by various factors, particularly in individuals with a genetic predisposition to sarcoidosis or autoimmunity [1].

The growing incidence and prevalence of sarcoidosis underscore its status as a significant global health issue, notably marked by varying rates among different ancestries, age groups, sexes, and geographic regions [2]. Accurately diagnosing sarcoidosis is complex, requiring a thorough consideration of multiple examinations to establish a comprehensive differential diagnosis. Whether the disease affects a single organ or multiple organs, various diagnostic tests and imaging modalities must be employed to diagnose it. For instance, an international study of 98 chest physicians and 48 thoracic radiologists, as part of the Delphi consensus study, determined that there are at least seven high-resolution computer tomography (HRCT) endophenotypes within pulmonary sarcoidosis [3]. This highlights the necessity for clinical and imaging findings to be consistent and substantiated by histological evidence of granulomas while excluding alternative causes. As sarcoidosis resembles many other diseases, clinical and imaging findings must be consistent and supported by histological evidence of granulomas, alongside the exclusion of alternative causes. Recently, a framework for differential diagnosis has been proposed by Harper et al. [4], which considers the heterogeneity of sarcoidosis and aims better to characterize the disease for improved and accurate diagnosis.

Given that sarcoidosis exhibits a variable clinical trajectory, the mortality rate and disability-adjusted life years (DALYs) Linked to pulmonary sarcoidosis constitute a significant global burden, demonstrating regional, demographic, and socioeconomic disparities across 204 countries [2]. Although many patients experience benign outcomes, approximately 50% encounter chronic complications, and 30% develop life-threatening conditions. Specifically, increased mortality is seen in patients who are older, have extensive fibrosis on HRCT scanning, or have pulmonary hypertension [5]. Neurologic and cardiac complications are common causes of mortality associated with sarcoidosis; however, respiratory failure remains the predominant cause of death related to sarcoidosis. A study from Spain, using the SARCOGEAS Registry, showed that renal, cardiac, and bone marrow involvement is associated with higher mortality compared to cutaneous, extrathoracic lymphadenopathy, and ocular involvement, which were associated with lower mortality [6]. Mortality studies related to sarcoidosis yield differing results contingent upon age, ethnicity, sex, and geographic location, highlighting the need for comprehensive analyses of mortality risks across diverse populations to understand better the factors influencing clinical outcomes in sarcoidosis.

Therefore, it is unequivocal that the heterogeneity inherent in sarcoidosis presents considerable diagnostic and therapeutic challenges. Although many cases resolve spontaneously, a significant subset of patients progress to chronic, progressive disease affecting the lungs, heart, nervous system, or other organs [7]. Current treatment modalities, such as corticosteroids, second- or third-line immunosuppressants, and biologics, are frequently prescribed empirically and are associated with substantial side effects, with no available curative options [8]. A recent pharmacovigilance study, utilizing data from the World Health Organization (WHO) and the VigiBase database, revealed 2,425 adverse drug reactions among patients with sarcoidosis [9], emphasizing the urgent need for improved management strategies.

Despite a decade of research and proposed treatment strategies [10], sarcoidosis remains a field with significant knowledge gaps, particularly in understanding the mechanisms that drive disease persistence, treatment resistance, and variability in clinical outcomes. Among the most underdeveloped yet promising areas are pharmacogenetics and pharmacogenomics, which offer the potential to bridge these gaps by identifying genetic and molecular markers that predict drug response, toxicity, and treatment outcomes.

Introducing pharmacogenetics and pharmacogenomics approaches in sarcoidosis could: 1) Personalize treatment selection, especially in patients with chronic or refractory disease; 2) Reduce adverse drug reactions by identifying at-risk individuals before therapy is initiated; 3) Guide optimal dosing strategies and improve treatment adherence; and 4) Enable stratification of patients in clinical trials based on molecular signatures of drug responsiveness.

The integration of multi-omics technologies, combined with clinical and treatment response data, presents an unprecedented opportunity to build predictive models of therapy outcomes. Yet, few studies have systematically explored these approaches in sarcoidosis, and no pharmacogenomic biomarkers have been validated for clinical use.

Given the disease's clinical heterogeneity, treatment challenges, and health disparities—especially among populations of African descent—sarcoidosis research urgently needs to embrace precision medicine tools, including pharmacogenetics and pharmacogenomics, to transform clinical care and improve patient-reported outcomes (PROs).

In this review, we focus on the vast knowledge acquired from genetics, including what is currently known about sarcoidosis phenotype characterization, areas for further improvement, and how genetics can contribute to therapeutic advances in precision medicine research, ultimately bridging the current knowledge gaps in the field.

## Genetic Insights into Sarcoidosis

As the genetic architecture of sarcoidosis is gradually being elucidated, progress is being made, and larger, more robust studies are contributing to this advancement. It is worth mentioning that, compared to the genetic investigations of other complex respiratory diseases, such as asthma and COPD, research on sarcoidosis has progressed at a slower pace. Over the past thirty years, genetic research in sarcoidosis has primarily involved small sample sizes and limited replication of findings across diverse populations and ancestries. This is mainly attributable to the insufficient funding allocated to this field. In addition to funding constraints, limitations in phenotype characterization have significantly hindered progress in genetic studies and, more recently, in omics developments. However, through extensive collaborative efforts, network initiatives, such as the MESARGEN Consortium and SARCOIDOMICS, as well as local and international gatherings, the sarcoidosis research community is uniting, fostering increased collaborations.

For more than two decades, researchers studying sarcoidosis have been trying to decipher the genetic underpinnings. What is known today is that the immunopathogenesis of sarcoidosis involves antigen presentation and granuloma formation, molecular mechanisms that involve Human Leukocyte Antigen (HLA) genes, including those in HLA class I, II, and III. Although class II mainly refers to the alleles of *HLA-DRB1*, which are polymorphic, and their alleles and frequencies vary tremendously across populations of different ancestries. Non-HLA genes have also been reported, which will be discussed later in the section.

The *HLA-DRB1* gene is located within the human major histocompatibility complex (MHC) class II region and plays a crucial role in the immune response. It encodes a protein responsible for presenting foreign peptides to immune cells, thereby initiating immune reactions [11]. For instance, in sarcoidosis, the breadth and specificity of CD4(+) T-cell responses are determined by *HLA DRB1*-specific epitopes in individuals with sarcoidosis susceptibility. Therefore, HLA-specific variations may serve as predictors of relationships between clinical outcomes and *HLA-DRB1* alleles. Notably, *HLA-DRB1* variations exhibit a significant association with susceptibility and clinical outcomes across a broad spectrum of autoimmune, infectious, inflammatory diseases, and cancer [12, 13].

The *HLA-DRB1* gene encodes a cell surface receptor that presents peptides to CD4 + T cells, initiating immune responses against pathogens and, in some cases, self-antigens. This gene is highly polymorphic, with different alleles influencing peptide binding and immune recognition, and is strongly associated with disease risk in various ethnic populations. The *HLA-DRB1* gene is closely linked; that is, in linkage disequilibrium (LD) (r^2^ > 0.8) to nearby genes in the same class II region. For instance, *HLA-DQA1* and *HLA-DQB1* are other HLA class II beta chain paralogs, which have also been reported to be associated with sarcoidosis in specific ethnic populations [14–17]. These class II molecules are heterodimers consisting of an alpha (DQA) and a beta chain (DQB), both of which are anchored in the cell membrane. These genes play a central role in the immune system by presenting peptides derived from extracellular proteins. Interestingly, these genes are also shared in endocrine autoimmune disease [18]. As *HLA-DRB1, HLA-DQ A1, and HLA-DQ B1* are expressed in antigen-presenting cells, i.e., B lymphocytes, dendritic cells, and macrophages, which are key cells in granuloma formation [19].

It is essential to note that *HLA-DRB1* not only acts in conjunction with genes in the vicinity but also interacts with other genes involved in immune regulation and response, as well as persistent inflammation across the genome. For instance, in an earlier study [1], we reported an interaction between *HLA-DRB1* and *ANXA11*. *ANXA11* is a crucial locus as it has been reported in various populations as a susceptible locus for sarcoidosis [20–23], and it is also linked to various sarcoidosis subphenotypes, particularly disease chronicity [24]. The product of the *ANXA11* gene is a calcium-dependent phospholipid-binding protein that plays a key role in the binding of N-terminal domains and conserved C-terminal domains, which contain calcium-dependent phospholipid-binding sites. Another gene–gene interaction of importance is between *HLA-DRB1* and *PTPN2*, which are both key susceptibility genes in anti-CCP–positive rheumatoid arthritis (anti-CCP + RA) [25]. Fascinatingly, *PTPN22* is believed to be a key master gene affecting various autoimmune diseases [26] as it is involved in the adaptive immune system pathway, which is linked with TCR signaling [27]. Another gene of great interest is *IL23R,* which has been reported to interact with cigarette smoking and disease chronicity in sarcoidosis patients [28, 29]. In recent GWAS in the Japanese population, *IL23R* was associated with ocular sarcoidosis [30]. Notably, *IL23R* is a well-known susceptibility locus for inflammatory bowel disease (IBD) [31]. It is posited that the different combinations of *HLA-DRB1* alleles and the interactions with master genes in adaptive immune response will generate a combinatorial peptide for antigen recognition, segmenting its role in chronic inflammation and autoimmunity.

**Fig. 1:**
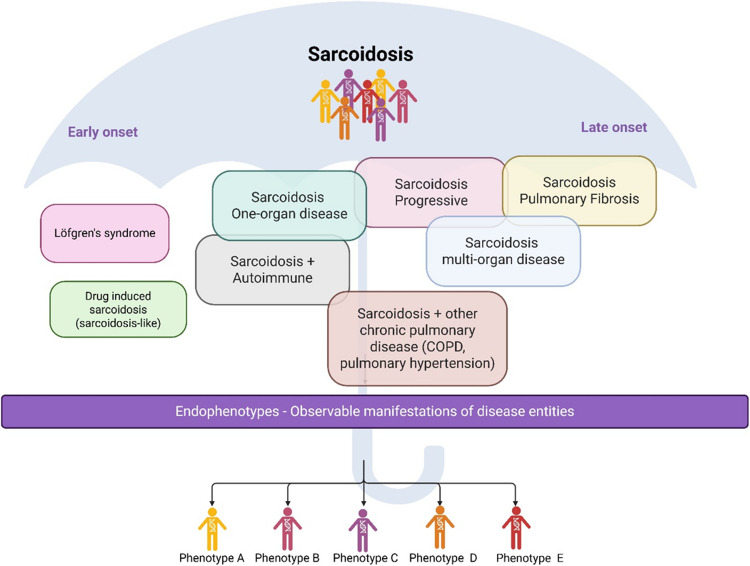
The genetic umbrella of sarcoidosis. Created with BioRender

As our understanding of sarcoidosis genetics advances, it appears that distinct phenotypes of the disease each have unique genetic architectures. These genetic differences likely play a significant role in shaping the diverse clinical presentations observed in patients. Figure 1 illustrates this idea by showing the analysis of various sarcoidosis subphenotypes alongside their associated genetic factors, emphasizing that specific patient groups may have genetic susceptibilities that influence their disease phenotype within the broader spectrum of sarcoidosis. Figure 1 illustrates this concept.

## Sarcoidosis Phenotypes and Disease Characterizations

### Phenotypes

Sarcoidosis is a highly variable disease in terms of presentation, evolution, outcome, treatment requirements, and prognosis. Many studies have been carried out with two main objectives in order to distinguish subgroups of patients based on their phenotypic characteristics. The first one is to design appropriate therapeutic strategies for the most homogeneous sarcoid patient groups as possible, bearing in mind that this approach will ultimately allow personalized therapeutic approaches. To apply to everyday clinical practices, phenotyping should be easy, reliable in detecting all organ involvement, and reproducible in all sarcoid patients, regardless of their geographic origin. These are the conditions for good treatment decisions based on individual phenotypes. The second goal of defining phenotypes here is for the sake of research, particularly genetic ones, to identify the true basis of sarcoidosis, which develops in a susceptible host after specific interactions with the environment.

a Radiology Phenotyping

The first identified sarcoidosis phenotypes were based on thoracic chest X-ray. Scadding described them into four stages: 1) enlarged hilar lymph nodes, 2) hilar nodes and lung shadowing, 3) lung shadowing, and 4) pulmonary fibrosis [32]. The same author also identified a particular phenotype, the Löfgren syndrome, with an extremely good prognosis.

The SARCOGEAS Study [6] aimed at analyzing the Scadding radiological stages of sarcoidosis at diagnosis with clinical features and epidemiology in a cohort of 1,230 patients from southern Europe. It showed that bilateral hilar lymphadenopathy, as well as the presence of fibrotic lesions, did not influence the systemic phenotype of pulmonary sarcoidosis and that the main element associated with a systemic involvement of sarcoidosis was the presence of interstitial pulmonary lesions rather than the individual Scadding stages.

b Clinical Tools for Phenotyping

A sarcoidosis assessment instrument was developed in the ACCESS study [33–35] and was subsequently upgraded by the WASOG (WASOG Sarcoidosis Organ Assessment Instrument) [36]. These tools were used, for instance, in the major GenPhenReSa study [37], carried out in a cluster analysis of 2163 Caucasian patients, which identified five subgroups characterized by different organ involvement: 1: abdominal, 2: ocular-cardiac-cutaneous-central nervous system, 3: musculoskeletal-cutaneous, 4: pulmonary and mediastinal lymph nodes, 5: extrapulmonary involvement [37]. The organ clusters observed in this European population were reproducible in an American one, with no difference between Black and White patients [38]. A French multi-ethnic and multicenter study (EpiSarc) confirmed the existence of distinct phenotypes in sarcoidosis, with a non-random distribution of organ involvement and clear associations between these phenotypes and socio-professional status, geographic origin, and sex [39]. Other studies focused on the potential association of clinical features and/or clusters and disease evolution and prognosis. That by Rubio-Rivas showed that the cluster model worked moderately well in terms of the prediction of chronicity [40].

iii.  Pulmonary Function Phenotyping

A mixed ventilatory defect, observed in about 25% of sarcoid patients, is associated with a stage 4 disease and has a higher mortality than a purely obstructive profile [41]. On the other hand, FEV1 and FVC are related to race and disease duration, while DLCO was related to male sex, race, and smoking [42].

iv. HRCT Phenotyping

Many attempts have been made to classify patients according to their imaging features, particularly after Scadding chest X-ray stages, CT scan, and PET-CT ones [43]. Quite recently, a multi-national Delphi study was carried out by Desai et al. [3] and a consensus was obtained on the stratification of likely to be fibrotic and non-fibrotic subtypes, with a 97% agreement on the existence of distinct HRCT phenotypes. The international consensus reached in this Delphi study highly justifies the CT classification as a basis for the possible definition of separate diseases.

e PET-CT Phenotyping

By detecting active inflammation in involved organs and particularly amongst fibrotic lesions in the lung, allowing the definition of active vs inactive sarcoidosis phenotype, PET-CT is bound to become key to a personalized therapeutic approach. It recently allowed the definition of 4 phenotypes according to the involvement of specific lymph node areas, lungs, and extrathoracic organs [44]. However, PET-CT is not yet recommended in the initial work-up of sarcoidosis, and further studies will have to determine its value as an outcome marker of these different phenotypes.

### Endophenotypes

Numerous biological markers of the different pathogenic pathways leading to the formation and persistence of granulomas are associated with distinct disease evolutive patterns and could be used as significant prognosticators. Table 1 highlights the review by Papanikolaou et al. [45], which summarizes the main biological actors involved in sarcoidosis in association with some of its different phenotypes.

**Table 1:** Characterization of endotypes of sarcoidosis. Adopted from Papanikolaou et al. [45]. Reproduced with permission from Papanikolaou et al., Biomedicines 2025; Published by MDPI

HLA-DRB1*03 HLA-DRB1*03, *0301 or *1501 HLA polymorphism (rs4143332) [46–49]	Associated with Löfgren syndrome
HLA-DRB1*07, *14, *15, *01 and *03 and DQB1*0602 [17, 47]	Higher likelihood of progressive pulmonary sarcoidosis
TNF-alpha polymorphisms (308AA and rs1800629) [50–52]	Pulmonary disease progression and acute-onset disease
*ANXA11* locus rs1049550 [53]	Protective effect of the minor T allele
Polymorphisms in TLRs (absence of the common haplotype in the *TLR10-TLR1-TLR6 *gene cluster, *TLR3 L412F*, *MyD88*, and *CybB/Nox2*	Chronic, persistent disease
*SEPP1* [54]	Worse lung function
*IL20RB, ABCC11, SFSWAP, AGB14, miR-146a-3p and miR-378b* in a multi-omics model [54]	Associated with progressive sarcoidosis
*miR-21-5p, miR-340-5p,* and *miR-212-3p* [55]	Differentiate patients with Löfgren syndrome
*miR-155, let-7c,* and transcription factor T-bet [56]	Progressive disease
Th1, Th17, IFN-gamma, and NFAT signaling (*CD128, STAT1, CXCR3* and *CCR4 *genes) [57]	Hilar lymphadenopathy
IL-2 and Il-7 pathways (*MRC2, SL40A1, F2R, IL7, PTPN7, ADORA2A, SPRY2, PLA2G7*, and *PTGS1*) [57]	More severe bronchial wall thickening
TGF-b1 and MTOR pathways (*TGFBR1, COL3A1, TLR3, ID1, TCF4,IGFBP6, PLAC2G7, FADS1, ARGHAP12* and *MMP10, SC5D,HIF1A*, and *PPAR-alpha*) [57]	Parenchymal involvement-pulmonary fibrosis
Four gene modules [57]	4 novel endophenotypes: chronic sarcoidosis, hilar lymphadenopathy and acute lymphocytic inflammation, multi-organ involvement with increased immune response, and extra-ocular involvement with PI3K activation
mTOR pathway activation [67,69,174] [58–60]	Sarcoidosis progression
JAK/STAT pathway activation (17-gene signature)[61]	Sarcoidosis progression
Increased Th1 and Th17.1 cells [62, 63]	Chronic sarcoidosis
Increased Th17 cells [64]	Löfgren syndrome
High expressiono f CD25, CTLA4, CD69, PD-1 and CD95 in blood Tregs [65]	Chronic sarcoidosis

The establishment of these biological profiles results from genetic, DNA methylome, transcriptomic, and proteomic studies [66] of bronchoalveolar lavage cells and fluid [67, 68] blood cells, plasma, and lung tissue. Quite recently, these proteomic techniques have been used to characterize the content of extracellular vesicles and have shown striking associations between some profiles and response to treatment (prednisone or methotrexate) [69].

Confirmation of these endotypes in large prospective cohorts will be a significant step in the delineation of personalized treatment strategies, as they will serve as key diagnostic and prognostic biomarkers as well as indicators of potentially new and more specific drug targets.

## Advances in Therapeutic Approaches

### Traditional Treatments and their Limitations

a Overview of current standard treatments

The last ERS guidelines for the treatment of sarcoidosis [70] were based upon expert opinions due to the lack at that time of any large prospective clinical trial. Corticosteroids (CS) were recommended as first-line agents with a starting dose of 20–40 mg/day and a more or less rapid tapering, aiming at limiting as much as possible the undesirable effects of the drug. CS has so far been the first-line agent, whatever the organ(s) involved and whatever the disease presentation, including those known as high-risk sarcoidosis [71–73]. The major second-line agent, to be introduced either because of persistence of the disease or sometimes early in the course of the disease as a CS sparing drug, is methotrexate. Again, no dose could be reliably recommended, but the usual one is 15 to 20 mg/week. Other second-line agents comprise Azathioprine, Leflunomide, Mycophenolate mofetil, and Hydroxychloroquine. Third-line agents are mainly TNF alpha inhibitors, whether infliximab or adalimumab. Finally, other drugs were only cited in the document [70] (JAK inhibitors, rituximab, repository corticotropin injections, with no recommendations as to their dose and duration.

Quite interestingly, recent publications have brought some answers to the underlying questions present in the ERS guidelines. For example, it has been shown that an initial dose of 20 mg of CS did as well as 40 mg on FVC and FEV1 improvement, as well as on health-related quality of life [74, 75]. A randomized, prospective study (PREDMETH) has also shown that methotrexate used upfront was equivalent to CS in terms of FVC improvement [76]. Considering the very different toxicity profiles of these 2 drugs, these data can now be part of a well-informed, shared decision between patients and caregivers about which drug to use at first line.

Also interestingly, more information is now available about biologic and targeted synthetic therapies used in sarcoidosis [77] as well as about some drugs only cited in the guidelines, such as JAK inhibitors, mainly tofacitinib, and mTOR inhibitors [78]. They will be described in more detail in the following chapter.

b Challenges and side effects associated with treatments

We will focus on the CS associated ones as they are the most frequently used drugs, generating the widest and most frequent undesirable effects. Because some of them are dose-dependent, the lowest dose possible is always recommended, as well as the use of CS sparing agents when a treatment of more than 6 months is anticipated. Finally, some of these toxic effects are related to preexisting comorbidities, which have to be carefully screened for before initiating the treatment and monitored throughout the disease course [79, 80].

Let's cite here the risk of generating or increasing a metabolic syndrome (weight gain, hyperglycemia (with a risk of diabetes type 2), hyperlipidemia, hypertension), gastritis, osteoporosis, adrenocortical insufficiency (appearing during tapering down CS), depression, ocular complications (glaucoma, cataract), increased risk of infection due to the marked immunodepression associated with a long course of CS. A comprehensive pretherapeutic work-up and precise monitoring of treated patients are therefore mandatory in order to minimize these risks and to design personalized patient care.

Table 2 highlights important parameters to monitor during the disease course.

**Table 2:** Summarizes all of the points of interest in this perspective. Adopted from Kwon et al. [80]. Reproduced with permission from Kwon et al., J Clin Med. 2024; Published by MDPI

Monitor parameter	Monitor time frame	Reference
Body weight	Baseline, frequently	[81]
Height	Baseline, annually	[81, 82]
Blood pressure	Baseline, frequently	[81, 83]
HbA1C	Baseline, every 3–6 months	[81]
Blood glucose	Baseline, frequently	[81]
CBC	Baseline, frequently	[81]
Lipid profile	Baseline, one month after initiation of glucocorticoid	[81, 83, 84]
Bone-mineral density	Baseline, every 1–2 years	[82]
Fracture history	Baseline, then routine follow-up visits	[81]
Joint pain	Baseline, then routine follow-up visits	[81, 85]
Eye exam	Baseline, then annually or as recommended by an ophthalmologist	[81, 83]
Healthy lifestyle inventory and education	Baseline documentation of the patient’s lifestyle and awareness. After initial counseling, reinforce healthy lifestyle choices at routine follow-up visits	[81]
Perceived fatigue	Baseline, then routine follow-up visits	[81, 86]
Adrenal insufficiency	Measure serum cortisol or perform an ACTH stimulation test in patients with symptoms of adrenal insufficiency (or withdrawal) who have been tapered to a low dose or off corticosteroids	[81]
Anginal symptoms (cardiovascular events)	Baseline, at routine follow-up visits, educate the patient concerning these symptoms	[81, 87]

*ACTH* adrenocorticotropic hormone

## Pharmacogenetics and Pharmacogenomics

Pharmacogenetics involves studying genetic variations, such as single-nucleotide polymorphisms (SNPs), that may influence drug response and contribute to adverse drug reactions. Understanding these genetic factors is crucial for clarifying individual differences in treatment effectiveness and toxicity, especially in complex inflammatory conditions like sarcoidosis [88]. Similarly, pharmacogenomics covers a broader scope, examining how an individual's genetic makeup across various biological layers—including the genome, proteome, and metabolome—affects drug metabolism, response, and interactions. This integrated approach is increasingly important in sarcoidosis research, as it aims to optimize personalized therapy, understand disease heterogeneity, and improve outcomes through targeted pharmacological strategies [89]. In this section, we will discuss a few relevant examples; however, this area of research remains an uncharted field of work in sarcoidosis, and more research is necessary.

As mentioned above, *HLA-DRB1* is a key gene linked to various subphenotypes in sarcoidosis, including target-organ involvement and disease persistence [24]. Tumor necrosis factor-alpha (TNF-alpha) is a critical cytokine involved in the pathophysiology of sarcoidosis [90]. Infliximab, a humanized chimeric monoclonal antibody, specifically binds to and neutralizes circulating TNF-alpha, thereby modulating inflammatory responses. From a pharmacogenetic perspective, specific *HLA-DRB1* alleles have been associated with variable responses to anti-TNF therapies across different immune-mediated diseases, highlighting the importance of genetic factors in treatment efficacy. For example, HLA-DRB1*03:01 or HLA-DRB1*04:04 is linked to a higher risk of drug-induced liver injury in response to infliximab [91]. HLA-DRB1 *04:01 is associated with increased response to adalimumab, etanercept, and infliximab in individuals with rheumatoid arthritis [92]. Infliximab is a third-line treatment for sarcoidosis [93], and it is often considered a therapeutic option in the subgroup of patients with refractory disease or patients who are intolerant to first and second-line agents [94]. Likewise, HLA-DQA1*15 has been linked to a lack of response to infliximab in inflammatory bowel disease (IBD) [95]. HLA-B*39:01 and HLA-DQB1*02:01 also showed increased risk for drug-induced liver toxicity [91]. From these examples, it is clear that other genetic and clinical factors may also influence the risk of drug-induced liver injury when treated with infliximab, indicating the need for further research, especially in sarcoidosis patients.

Another essential gene to consider is *CTLA-4*, which has been linked to the development of pulmonary and cutaneous sarcoidosis in patients treated with anti-CTLA-4 and anti-PD-L1 therapies for metastatic melanoma [96, 97]. For instance, ipilimumab has been reported to be linked with several immune-related adverse reactions, including uveitis and pulmonary sarcoidosis-like reaction (98). Patasova et al. identified shared genomic loci between cancer and sarcoidosis, suggesting common genetic factors [99]. Additionally, Broos et al. observed decreased expression of *CTLA-4* on regulatory T cells (Tregs) and Th17 cells in sarcoidosis patients, particularly in mediastinal lymph nodes [100]. These findings suggest a shared genetic susceptibility and immune regulation mechanisms between cancer and sarcoidosis, emphasizing the role of immune-mediated genes involved in targeted therapy and their importance in both conditions.

As genetic research and phenotypic characterization in sarcoidosis advance, new opportunities will emerge to identify genetic variants and molecular profiles that influence individual responses to pharmacological treatments. Through pharmacogenomic studies, we will be able to uncover biomarkers associated with drug efficacy and adverse reactions, paving the way for personalized therapeutic strategies. Ultimately, integrating genetic insights into clinical decision-making will enhance precision medicine approaches for patients with sarcoidosis and related immune-mediated conditions.

## Future Directions

The molecular mechanisms underlying the pathogenesis of sarcoidosis and the factors driving its clinical heterogeneity remain significant knowledge gaps with important clinical implications. As aforementioned, sarcoidosis encompasses a broad spectrum of clinical phenotypes, influenced in part by genetic predisposition, ranging from asymptomatic or self-limiting presentations to chronic, progressive disease with potentially life-threatening involvement of organs such as the heart and nervous system. To advance precision medicine in sarcoidosis, the research community must prioritize efforts to close these knowledge gaps through several key directions that we strongly emphasize:

### Unmet Clinical Needs

 Current treatment strategies rely heavily on non-specific immunosuppression, often guided by trial and error. Many patients experience suboptimal responses, drug-related toxicity, or long-term complications from chronic inflammation and fibrosis. Developing more effective, targeted therapies is essential.

### Lack of Biomarkers

There remains an urgent need for reliable diagnostic, prognostic, and therapeutic biomarkers that can guide clinical decision-making, monitor disease activity, and predict treatment response.

### Health Disparities

Sarcoidosis disproportionately affects individuals of African descent, who often have more severe and chronic disease courses. Understanding the genetic, environmental, and social determinants that contribute to these disparities is crucial for equitable care.

### Broader Relevance

Studying sarcoidosis offers broader insights into granulomatous inflammation, immune regulation, and tissue remodeling, with implications for other immune-mediated and fibrotic diseases.

### Pharmacogenetics and Pharmacogenomics

One of the most underdeveloped but promising areas is the integration of pharmacogenetic and pharmacogenomic strategies. These approaches have the potential to revolutionize sarcoidosis treatment by identifying molecular predictors of drug response and toxicity, thereby enabling the development of personalized therapies. Systematic efforts to link genomic data with treatment outcomes and adverse effects are essential for optimizing therapeutic efficacy and minimizing harm.

### Leveraging Multi-Omics and Systems Biology

Advances in genomics, transcriptomics, proteomics, and epigenomics offer unprecedented opportunities to decode the complex immune dysregulation and granuloma biology that characterize sarcoidosis. Integrating multi-omics data with deep clinical phenotyping and expanding to pharmacogenomics can help uncover disease mechanisms and identify new therapeutic targets.

Addressing these challenges through coordinated, interdisciplinary research will be critical to transforming sarcoidosis from a disease of empirical management to one guided by molecular precision.

## Conclusion

To advance personalized treatment strategies for sarcoidosis, it is essential to continue genetic research with large sample sizes across various ethnic populations and deep-phenotyping for dissecting the underlying disease heterogeneity in sarcoidosis. To facilitate the advances of precision medicine efforts, we must keep in mind the integration of pharmacogenetics and pharmacogenomics in sarcoidosis research. These methods can identify new drug targets tailored to patients' specific genetic susceptibility. Additionally, accurate phenotyping remains a critical challenge; deep phenotyping can help us define genetic factors associated with different phenotypes within the broader spectrum of sarcoidosis and other immune-mediated disorders, which will improve our understanding of the genetic umbrella of sarcoidosis. Addressing these areas will lead to more targeted and effective therapies, ultimately enhancing patient outcomes.

## Data Availability

No datasets were generated or analysed during the current study.
